# Fate of Antibiotic Resistance Genes and Changes in Bacterial Community With Increasing Breeding Scale of Layer Manure

**DOI:** 10.3389/fmicb.2022.857046

**Published:** 2022-03-09

**Authors:** Lixiao Wang, Baofeng Chai

**Affiliations:** Shanxi Key Laboratory of Ecological Restoration for Loess Plateau, Institute of Loess Plateau, Shanxi University, Taiyuan, China

**Keywords:** antibiotic resistance genes, intensive layer breeding, manure, pathogenic bacteria, breeding scale

## Abstract

The use of antimicrobials in intensive poultry production is becoming increasingly common because of its high throughput of meat and egg products. However, the profile of antibiotic resistance genes (ARGs) and the underlying mechanisms in different breeding scale farms were not fully explored. The study examined the profiles of ARGs in layer manure from three free-range and 12 intensive layer farms with different scales (N500, N5000, N10000, and N20000). A quantitative PCR (qPCR) array was used to quantify ARGs, and microbial community structure was analyzed by 16S rRNA gene sequencing. A total of 48 ARGs, belonging to seven major types, were identified in the layer manure samples, with *sul*2, *tet*M-01, and *erm*B being the predominant ones. The abundance, diversity, and mobility potential of ARGs in layer manure changed significantly with the increasing of the breeding scale. The abundances of total ARGs had significantly positive correlations with mobile genetic elements (MGEs), suggesting the mobility potential of ARGs in layer manure samples. Bacterial abundance did not show significant differences among the five group manure samples. However, bacterial diversity showed an increasing trend along the breeding scale. Pathogenic *Bacteroidetes* increased in the largest-scale layer manure samples and showed significant positive correlations with most ARGs. Network analysis revealed significant co-occurrence patterns between ARGs and microbial taxa, indicating ARGs had a wide range of bacterial hosts. Proteobacteria and Firmicutes were potential hosts for tetracycline and macrolide-lincosamide-streptogramin B (MLSB) resistant genes. Our results indicated that the expansion of the breeding scale of a farm promotes the abundance, diversity, and mobility potential of ARGs in layer manure.

## Highlights

- Antibiotic resistance genes in layer manure changed significantly with the increasing of breeding scale.
- Mobile genetic elements increased in the largest scale layer manure samples.
- Bacterial diversity showed an increasing trend along the breeding scale.
- Pathogenic *Bacteroidetes* showed significant positive correlations with most ARGs.

## Introduction

Livestock and poultry breeding, as the pillar industry of agriculture, plays a vital role in improving the living standards of the population (Xia et al., 2017). With the intensive development of the breeding industry, antibiotics are often added to feed to promote animal growth or improve animal immunity (Qian et al., 2018). The Chinese government implemented Animal Medicine Management Regulations in 2004. However, more than 84,000 tons of antibiotics were consumed by animals in 2013, which accounted for 52% of total antibiotic usages (Zhang et al., 2015). The abuse of antibiotics could induce the occurrence of antibiotic resistance genes (ARGs) in animal gut (Rodriguez-Rojas et al., 2013; Hu et al., 2014). Most of the antibiotics used in humans or poultry are not fully metabolized, and they are discharged into the external environment through the application of manure, soil leakage, surface run-off, and escape into the atmospheric particulate matter (Udikovic-Kolic et al., 2014; McEachran et al., 2015). But more disturbingly, through the aid of mobile elements such as plasmid and integron, ARGs could be incorporated into crops and enter human bodies through the food chain, further endangering public health (Wen et al., 2019). Driven by the “One Health” concept, which emphasizes the interdependence of human, animal, and environmental health, ARGs are regarded as emerging environmental pollutants and are a major public health concern (Pruden et al., 2006; Sanderson et al., 2016), especially when found in pathogens infecting humans (Jiang et al., 2017). Further, the emergence in recent years of “superbugs” with multidrug resistance highlights the seriousness of bacterial resistance in the livestock breeding environment (Nagarajan et al., 2018, Gu et al., 2020).

At present, ARG pollution in livestock waste has attracted widespread and increasing attention from scholars. Zhu et al. (2013) analyzed the pig farm environment of large-scale commercial farms in Beijing, Zhejiang, and Fujian provinces, including the presence of heavy metals and antibiotics in animal manure and soil. They used high-throughput fluorescence quantitative sequencing technology to analyze 244 kinds of resistance genes in environmental samples and finally found 149 resistance genes. Xie et al. (2016) determined the abundances of 213 ARGs and 10 marker genes for mobile genetic elements (MGEs) in commercial composts made from cattle, poultry, and swine manures in Eastern China, and they found that fresh poultry manure had the highest diversity of ARGs (137 genes detected), followed by fresh cattle manure (121 genes detected). Zhou et al. (2020) detected 109 kinds of ARGs in manure samples from large-scale farms of chickens, pigs, and cattle, and the pollution level of ARGs in chicken farms and pig farms was significantly higher than that in cattle farms. Meantime, researchers detected that these genes cover all the known antibiotic resistance types. All the above proves that livestock and poultry manure is an important reservoir of ARGs (Guo et al., 2017). A study also found that ARGs and MGEs were much more abundant in production chickens than in household chickens (up to a 157-fold difference; Guo et al., 2018). Furthermore, the environmental diffusion of antibiotics may influence selection pressure on bacterial communities and thus the abundance of ARGs (Han et al., 2018; Chen et al., 2019). Although commensal bacteria are normally harmless, they may constitute a reservoir of resistance genes that may be transmitted to pathogenic bacteria.

To meet the increasing demand for meat and egg products, more and more farms have gradually evolved into intensive farms with high-density methods, a large number of animals, a short feeding cycle and a rapid listing of animals. The epidemic caused by pathogenic microorganisms has become the biggest risk faced by the breeding industry. Our concern is whether the microbial community structure and diversity and the abundance and diversity of ARGs are also changing with the increase of breeding scale. As already reported, significantly higher levels of antibiotic resistance in *Escherichia coli* isolates in farmed production chickens vs. household chickens raised within the same communities (Guo et al., 2018). According to statistical analysis, the number of animals on farms and animal species explained differences in ARG abundance in manure samples (Muurinen et al., 2017). There are still many gaps in our knowledge about ARG profiles in different breeding farms, especially regarding the ecological mechanism of the microbial community for ARG spread. As is widely known, bacteria are the reservoir and transfer vector of ARGs. Therefore, studying the relationship between ARGs and the bacteria community is helpful for clarifying the ecological mechanism of the spread and distribution patterns of ARGs, thus reducing the health risks. In the present study, we investigate the abundance and diversity of ARGs and the bacterial communities in different breeding scales of layer farms. We aimed to assess (1) the impact of breeding scales on the ARG/MGE profile and bacteria community of layer manure samples, and (2) the relationship between ARGs and bacterial communities. The results should provide useful baseline information regarding ARGs’ prevalence from their source in layer farms.

## Materials and Methods

### Study Area and Sample Collection

In May 2020, we collected 15 manure samples of layers from 15 farms in Lvliang city of Shanxi province, China. For each sample, five isolated manure droppings from the ground were picked and mixed to form a combined sample. All samples were divided into five groups (each group contains three samples from different farms), including CK, N500, N5000, N10000, and N20000. The CK group represented free-range farms that kept about 200 layers. Group N500, N5000, N10000, and N20000 are intensive farms, and the layer breeding scales are 500, 5,000, 10,000, and 20,000, respectively. Before sampling, the breeding years and antibiotic use of the selected farms were investigated. The breeding years of the farms are more than 5 years. The 15 farms in the research use the veterinary drugs from the same company, and the single dosage of veterinary drugs were consistent.

Manure samples were collected by a sterile manure sampling tube, stored in an ice box, delivered to the laboratory within 24 h, and stored in an ultra-low temperature freezer (−80°C) for DNA extraction.

### DNA Extraction

Microbial community genomic DNA was extracted from manure samples using the E.Z.N.A.® soil DNA Kit (Omega Bio-tek, Norcross, GA, United States) according to the manufacturer’s instructions. The DNA extract was checked on 1% agarose gel, and DNA concentration and purity were determined with a NanoDrop 2,000 UV-vis spectrophotometer (Thermo Scientific, Wilmington, United States).

### ARGs and MGEs Analysis by Quantitative PCR

Quantitative PCR (qPCR) was applied to identify 48 target ARGs that exhibit resistance to the seven most commonly used types of antibiotics (the primers are shown in Supplementary Table S1). The seven target groups are as follows: aminoglycosides, β-lactamases, fluoroquinolones, quinolones, florfenicols, chloramphenicols, and amphenicols (FCAs), MLSBs, sulphonamides, tetracyclines, and other/efflux. The ARGs examined in this study are as follows: *aac*(6′)-IB-1, *aac*(6′)-II, *aac*A/aphD, *aad*A-02, *aad*A1, *aad*A2-03, *aad*D, *aad*E, *aph*A1, and *aph*A3-01 (aminoglycoside genes); *bla*CTX-M-02, *bla*OXA1/*bla*OXA30, *bla*OXA10-01, *bla*PSE, *bla*TEM, and *cfx*A (β-Lactamases genes); *cat*B3, *cfr*, and *flo*R (FCAs genes); *erm*(35), *erm*B, *erm*C, *erm*F, *erm*T-01, *lnu*A-01, *lnu*B-01, *mat*A/*mel*, and *mef*A (MLSBs genes); *sul*1 and *sul*2 (sulphonamides genes); *tet*(32), *tet*(36)-01, *tet*B-01, *tet*G-01, *tet*H, *tet*L-02, *tet*M-01, *tet*O-02, *tet*PA, *tet*PB-03, *tet*Q, *tet*R-03, *tet*T, *tet*W-01, and *tet*X (tetracyclines genes); *cat*B8 and *dfr*A1 (other/efflux genes). The transposon-transposase gene (*tnp*A-07) and integron-integrase gene (*int*I1) were also quantified as indicators for the horizontal gene transfer potential of ARGs. The relative abundance was assessed by the widely used method of 2^−△CT^ shown in Eq. 1 (Schmittgen and Livak, 2008).


(1) $$\Delta C_{T}=C_{T,\left(\mathrm{ARGs}\right)}–C_{T,\left(16S\;\mathrm{rRNA}\right)}$$

The C_T,(ARGs)_ and C_T,(16s rRNA)_ were the amplification threshold cycles, which stand for ARG subtypes and 16S rRNA genes from PCR experiments. In this study, the reference was threshold cycles for 16S rRNA, the standard curves of 16S rRNA was also established for quantitative analysis. The copy number of 16S rRNA gene was used to calculate the relative and absolute abundance of ARGs in manure samples.

Quantitative PCR analysis was performed by Wcgene Biotechnology (Shanghai) using the Applied Biosystems ViiA 7 Real-Time PCR System. A total of 10 μl of qPCR mixture was prepared with 5 μl of TB Green Premix Ex Taq II (Tli RNaseH Plus; 2×), 1 μl of template, 0.4 μl of each primer, 3 μl of ddH_2_O and 0.2 μl of ROX reference dye (50×). Reaction conditions were as follows: denaturation at 95°C for 10 min, followed by 40 cycles of 95°C for 30 s and annealing at 60°C for 30 s. A final melting curve was generated, ranging from 60 to 95°C, to determine the specificity of amplification. The limitation of the threshold cycle (Ct) was 40 in this study.

### Illumina Sequencing Analysis

The hypervariable region V3–V4 of the bacterial 16S rRNA gene was amplified with primer pairs 338F (5′-ACTCCTACGGGAGGCAGCAG-3′) and 806R (5′-GGACTACHVGGGTWTCTAAT-3′) by an ABI GeneAmp® 9700 PCR thermocycler (ABI, CA, United States). The PCR amplification of the 16S rRNA gene was performed as follows: initial denaturation at 95°C for 3 min, followed by 27 cycles of denaturing at 95°C for 30 s, annealing at 55°C for 30 s, extension at 72°C for 45 s, single extension at 72°C for 10 min and ending at 4°C. Purified amplicons were pooled in equimolar and paired-end sequenced on an Illumina MiSeq PE300 platform/NovaSeq PE250 platform (Illumina, San Diego, United States) according to the standard protocols by Majorbio Bio-Pharm Technology Co. Ltd (Shanghai, China). The observed taxonomic unit (OTU) was defined at a 97% similarity level using UCLUST. The raw 16S rRNA gene sequencing reads were demultiplexed, quality-filtered by fastp version 0.20.0 and merged by FLASH version 1.2.7 with the following criteria: (1) the 300 bp reads were truncated at any site receiving an average quality score of <20 over a 50 bp sliding window; the truncated reads shorter than 50 bp were discarded, and reads containing ambiguous characters were also discarded. (2) Only overlapping sequences longer than 10 bp were assembled according to their overlapped sequence. The maximum mismatch ratio of the overlap region is 0.2. Reads that could not be assembled were discarded. (3) Samples were distinguished according to their barcode and primers, the sequence direction was adjusted, exact barcode matching was confirmed and the two nucleotide mismatch in the primer matching was removed. Operational taxonomic units with 97% similarity cutoff value were clustered using UPARSE version 7.1, and chimeric sequences were identified and removed. The taxonomy of each OTU representative sequence was analyzed by RDP Classifier version 2.2 against the 16S rRNA database using a confidence threshold of 0.7.

### Statistical Analysis

The significance level of ARGs, MGEs, and 16S rRNA genes among the sample sites were performed by one-way ANOVA. A model based on regression analysis was created to assess the relationship between ARGs and MGEs. A CI of 95% was considered to be statistically significant, and statistical analyses and Pearson correlation tests were performed using SPSS 24.0 (IBM SPSS statistics, Chicago, IL, United States). Non-metric multidimensional scaling (NMDS) analysis results were plotted using the ggplot2 R package with ellipses indicating the 95% confidence region of each cluster. The similarity of the ARG profiles of manure samples between five group farms was tested by the NMDS and Analysis of similarities (ANOSIM) methods. The alpha diversity indexes of the bacterial communities were calculated in the R package Vegan. The co-occurrence network analyses, which represented the possible pairwise correlations between the ARGs and major bacterial taxa, were visualized in Gephi 0.9.2. Co-occurrence network analyses were calculated according to the results of possible pair-wise Spearman’s rank correlations.

## Results

### Abundances of ARGs and MGEs Change With the Breeding Scales

**Figure 1:**
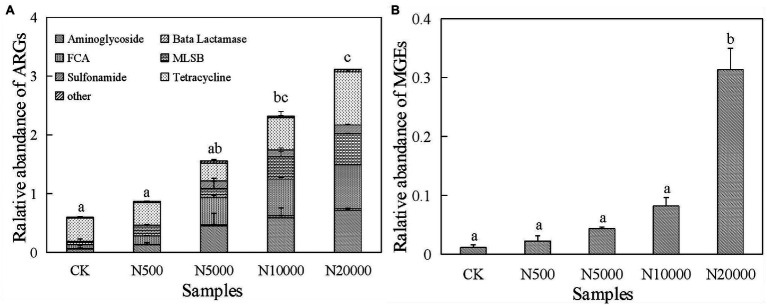
Relative abundance of antibiotic resistance genes (ARGs; **A**) and mobile genetic elements (MGEs; **B**) in manure samples. Different lowercase letters indicated significant differences (ANOVA, *p* < 0.05) between manure samples.

Forty-eight ARGs were detected in layer manure from 15 layer farms with different breeding scales. The relative abundance of ARGs within their bacterial community was represented by their proportion relative to the 16S rRNA gene for each layer manure sample. The relative abundance of ∑ARGs in the layer manure was ranged from 0.47 to 4.30 copies/16S rRNA gene copies. These ARGs covered almost all the major classes of antibiotics that are administered commonly to humans and animals, including aminoglycoside, β-lactamase, FAC, MLSB, sulphonamide, tetracycline, and other types of resistance genes. We observed the tetracycline genes to be the most frequently detected ARGs in the layer manure samples, followed by those conferring resistance against FAC, MLSB, and aminoglycoside (Figure 1A; Supplementary Table S2). The predominant ARGs of layer manure in intensive farms were the sulphonamide resistance gene *sul*2, the tetracycline resistance gene *tet*M-01, and the MLSB resistance gene *erm*B, with the relative abundance ranging from 5.31 × 10^−3^ to 2.57 × 10^−1^, 3.53 × 10^−2^ to 4.41 × 10^−1^, and 1.62 × 10^−2^ to 3.70 × 10^−1^ copies/16S rRNA gene copies, respectively. The dominant ARG in the free-range farms was the tetracycline resistance gene *tet*W-01, with the relative abundance ranging from 2.30 × 10^−1^ to 3.82 × 10^−1^ copies/16S rRNA gene copies. As for all the manure samples in layer farms, the relative abundance of ∑ARGs was significantly lower in the free-range farms than in the intensive farms, and it was significantly increased in the largest scale farms (N20000). MLSB and tetracycline taxa of ARGs were significantly different in the manure of each of the five group farms (*p* < 0.05), whereas aminoglycoside, FAC, sulphonamide, β-lactamase, and other types of ARGs had no significant difference.

The relative abundance of MGEs in manure samples was significantly increased in the group N20000 (*p* < 0.05; Figure 1B). The *tnp*A-07 gene and the intI1 gene were found in all the layer manure samples, and the *tnp*A-07 gene was the predominant MGE, ranging from 3.09 × 10^−3^ to 4.72 × 10^−1^ copies/16S rRNA genes, whereas the significant difference was found between group N20000 manure samples and the other samples. The other subtype of MGEs (*int*I1) within each group were not significantly different because they have a lower abundance (Supplementary Table S3).

### Correlation Among ARGs and MGEs

**Figure 2:**
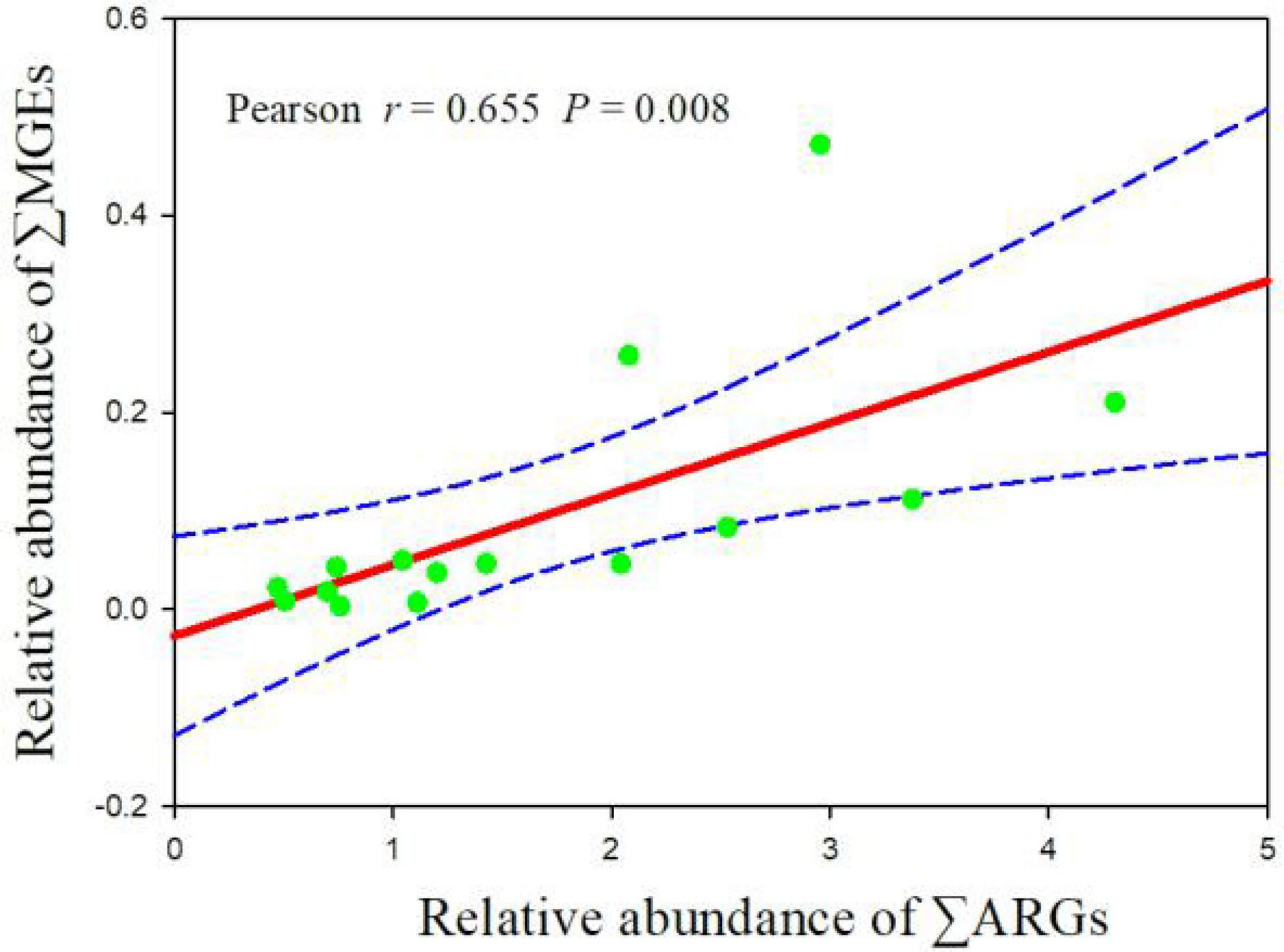
Correlation between the relative abundance of total ARGs (∑ARGs) and total MGEs (∑MGEs). Total ARGs and total MGEs are the sum of the relative abundance values of all assays of that type in each sample.

Pearson correlation analysis was performed to assess the correlations between ARGs and MGEs in manure samples (Figure 2; Supplementary Table S4). The results showed that the relative abundance of ∑ARG correlated significantly with the relative abundance of ∑MGE (*p* = 0.008, *r* = 0.655), indicating that MGEs may play a crucial role in the dissemination of ARGs.

For all samples, the *tnp*A-07 gene showed significantly positive correlations with *erm*B, *erm*T-01, *lun*A-01, *tet*L-02, and *tet*M-01 (*p* < 0.01) as well as being positively correlated with *aac*(6′)-IB-1, *aac*(6′)-II, *aad*A-02, *aad*A1, *aad*A2-03, *cat*B3, and *erm*C (*p* < 0.05). *Int*I1 was significantly positively correlated with *sul*1, *tet* (32), *tet*PA, and *tet*O-02 (*p* < 0.01), and it was also positively correlated with *cfr*, *erm*C, and *tet*PB-03 (*p* < 0.05). In the intensive farms, the *tnp*A-07 gene showed significantly positive correlations with *erm*B, *lun*A-01, *tet*L-02, and *tet*M-01 (*p* < 0.01), and it was also positively correlated with *erm*T-01 (*p* < 0.05). *Int*I1 was significantly positively correlated with *sul*1, *tet*(32), *tet*PA, and *tet*O-02 (*p* < 0.01), and it was also positively correlated with *erm*C and *tet*B-01 (*p* < 0.05). In the free-range farms, the *tnp*A-07 gene showed positive correlations with *erm*T-01 (*p* < 0.05). *Int*I1 was significantly positively correlated with dfrA1 and floR (*p* < 0.01), and it was also positively correlated with *aad*D, *bla*OXAI/*bla*OXA30, and *sul*1 (*p* < 0.05).

### Differences of the ARG Profiles in the Layer Manure Among the Different Scale Farms

**Figure 3:**
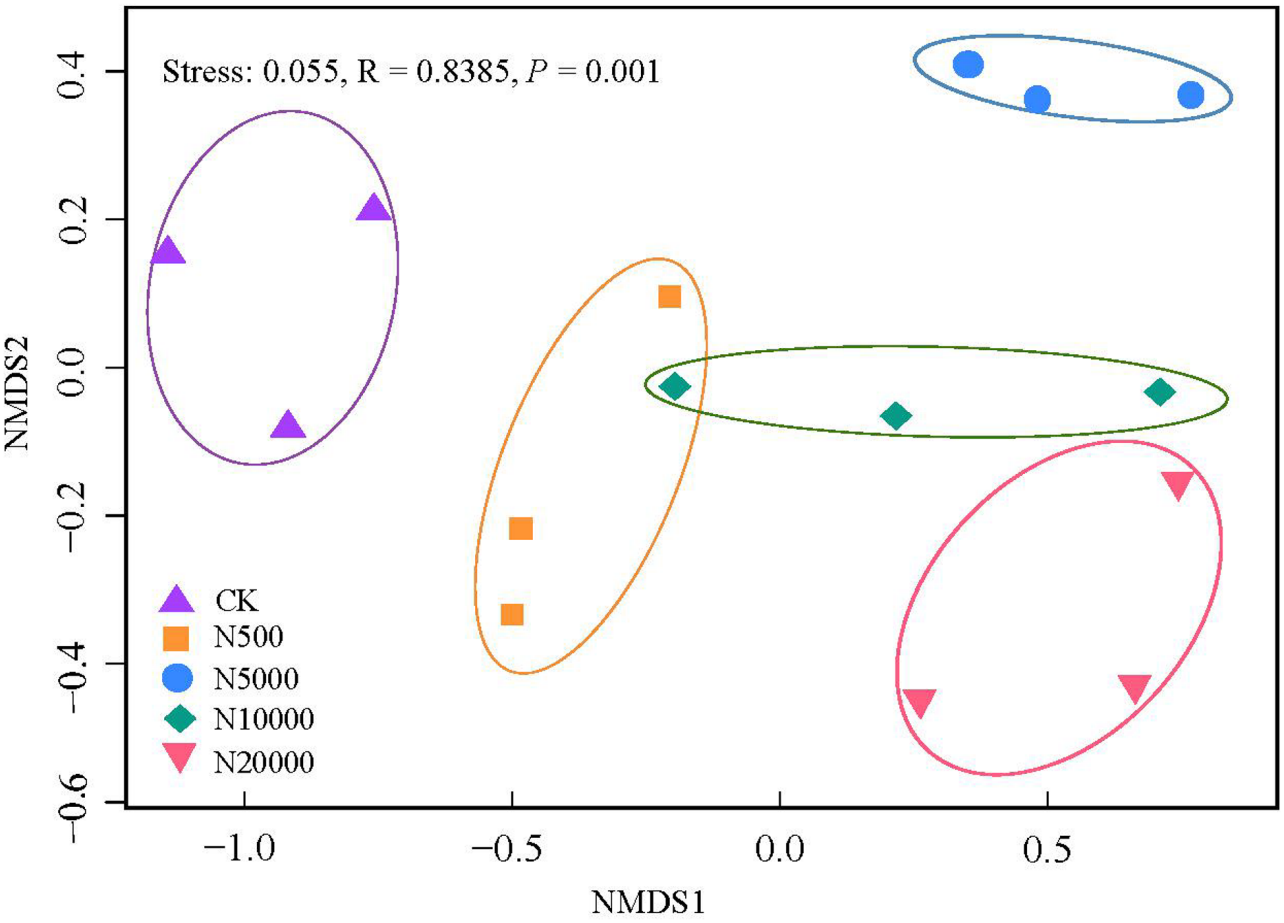
Non-metric multidimensional scaling (NMDS) ordinations derived from the Bray–Curtis dissimilarity matrices showing the similarity of the antibiotic resistance gene profiles of the manure between the different group farms. The stress value is lower than 0.10, indicating that the data are well represented by the two-dimensional ordinations.

Non-metric multidimensional scaling analysis using the Bray–Curtis distance derived from the relative abundance of ARGs was used to explore layer manure sample clustering based on the different farms. The ellipses indicate the 95% confidence region of each cluster. Given that the stress value was 0.05 in this study, the results of the NMDS analysis were considered well representative. The results revealed a separation of five group farms (ANOSIMR; *p* < 0.001; Figure 3), indicating that there were significant differences in the antibiotic resistance gene composition among the different breeding scales.

### Bacterial Communities in the Layer Manure in Farms With Different Breeding Scales

A total of 659,492 high-quality sequences were acquired from all layer manure samples, with 34,810–55,444 sequences per sample. Most of the filtered reads were approximately 415–428 bp in length, and rarefaction curves were shown in Supplementary Figure S1. These sequences were clustered into OTUs at a 97% similarity level, with an average of 1,289 OTUs per sample.

Bacterial abundance measured by qPCR, expressed as gene copies per gram of layer manure, ranged from 1.44 × 10^6^ to 3.03 × 10^6^ 16S rRNA copies/g of all manure samples. The bacterial 16S rRNA gene copy number was not significantly different across the five group samples (Supplementary Figure S2). The results showed that there was no difference in the abundance of bacterial community among the five group manure samples.

According to the analysis of bacterial alpha diversity, the Shannon values demonstrated a higher bacterial diversity of manure in the intensive farms (N500, N5000, N10000, and N20000) compared with that in the free-range farms (CK). The OTUs, ACE, Chao1, and Shannon indices were all minimal in the free-range farms compared to the intensive farms, and the bacterial community richness showed an increasing trend along the direction of the breeding scale (Table 1).

**Table 1:** Diversity differences of the bacterial communities in layer manure samples.

	OTUs	Shannon	Simpson	ACE	Chao1
CK	251.00 ± 200.00b	1.84 ± 1.11b	0.41 ± 0.27a	362.93 ± 187.30c	331.27 ± 195.68b
N500	427.67 ± 310.90ab	3.44 ± 1.27a	0.11 ± 0.08b	592.86 ± 287.72abc	546.21 ± 324.64ab
N5000	362.67 ± 137.06ab	3.53 ± 0.47a	0.07 ± 0.03b	478.91 ± 201.59bc	464.15 ± 180.86ab
N10000	630.00 ± 38.93a	3.67 ± 0.41a	0.12 ± 0.06b	771.94 ± 30.29ac	763.92 ± 39.86a
N20000	702.00 ± 118.20a	4.75 ± 0.33a	0.02 ± 0.01b	814.44 ± 129.97a	828.54 ± 132.71a

*Data in the table are mean ± SD. Sample number *n* = 3. Different lowercase letters within rows indicate significant difference at the *p* < 0.05 level*.

**Figure 4:**
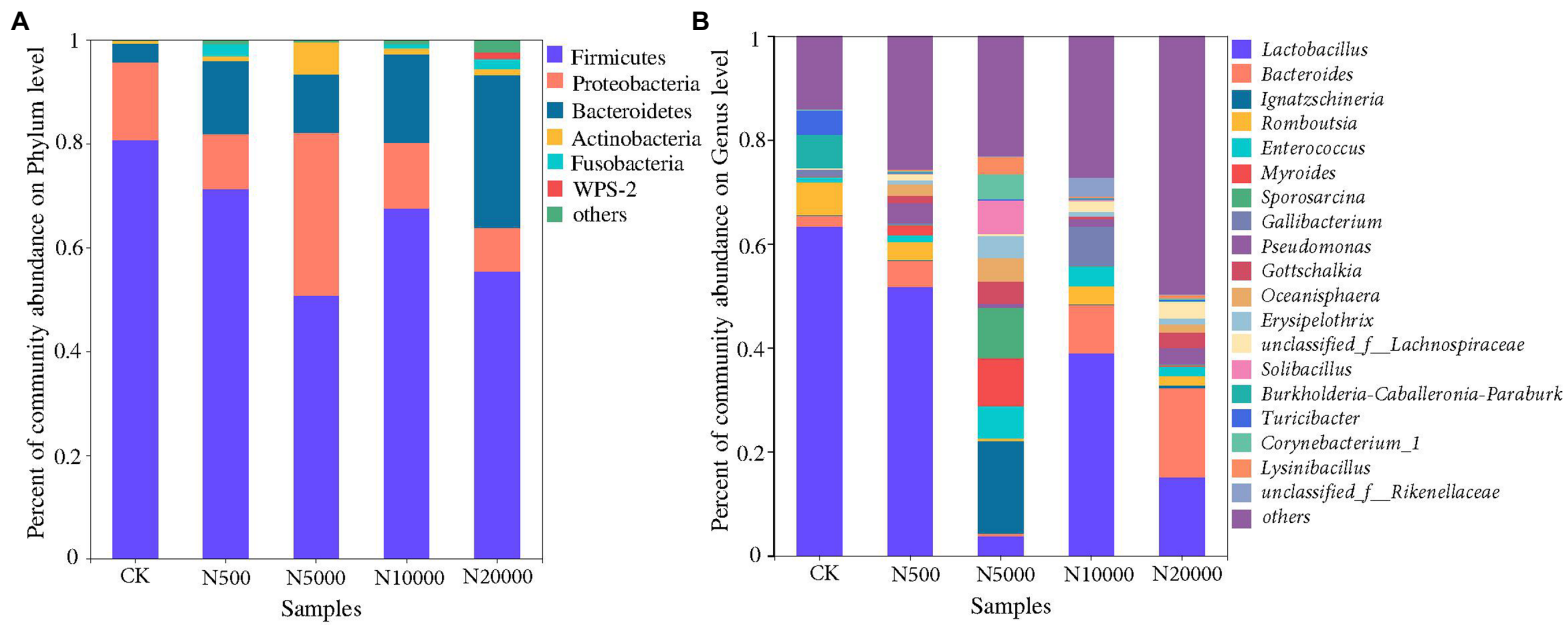
The bacterial community composition at the phylum level **(A)** and genus level **(B)** in different layer manure samples.

The composition and percent of bacterial communities varied greatly in the different manure sample classifications. According to the results of bacterial community abundance based on phylum analysis (Figure 4A), Firmicutes was the most dominant phylum in all layer manure samples, which accounted for 80.69, 71.28, 50.77, 67.53, and 55.39% of the total reads, respectively. Proteobacteria and Bacteroidetes were the next most abundant phylum in all layer manure samples, which ranged from 8.38 to 31.37% and 3.40 to 29.42%, respectively. In general, the Firmicutes, Proteobacteria, Bacteroidetes, Actinobacteria, and Fusobacteria consisted of the indigenous microbial communities of the manure in layer farms. The abundance of Proteobacteria and Epsilonbacteraeota showed a significant difference among the five group manure samples (ANOVA, *p* < 0.05; Supplementary Figure S3A). According to the results of bacterial community abundance based on genus analysis (Figure 4B), the most abundant genus was *Lactobacillus* in all layer manure samples, with the percentage of *Lactobacillus* community abundance ranging from 3.84 to 63.34% of the total reads. The abundance of *Bacteroides*, *Myroides*, *Pseudomonas*, *Gottschalkia*, and *Erysipelothrix* showed significant differences among manure samples from different scale farms (ANOVA, *p* < 0.05; Supplementary Figure S3B). It was noted that pathogenic bacteria had been detected in manure samples, whereas *Bacteroides* (0.51%a significant difference–17.12%) and *Enterococcus* (1.09–6.19%) were found to be the two main dominants (Figure 4B). *Campylobacter*, *Bacillus*, *Corynebacterium*, *Yersinia*, *Helicobacter*, *Burkholderia-Paraburkholderia*, *Staphylococcus*, *Acinetobacter*, *Escherichia-Shigella*, *Brucella*, and *Clostridium_sensu_stricto* were also detected with relatively low levels but were demonstrated to be harmful regarding their ability to cause human diseases. Significantly, *Bacteroidetes* increased in the largest scale (N20000) layer manure samples (*p* < 0.05; Supplementary Figure S3B), indicating that pathogenic bacteria may increase with the increase of breeding scale.

### Potential Bacterial Hosts of ARGs

**Figure 5:**
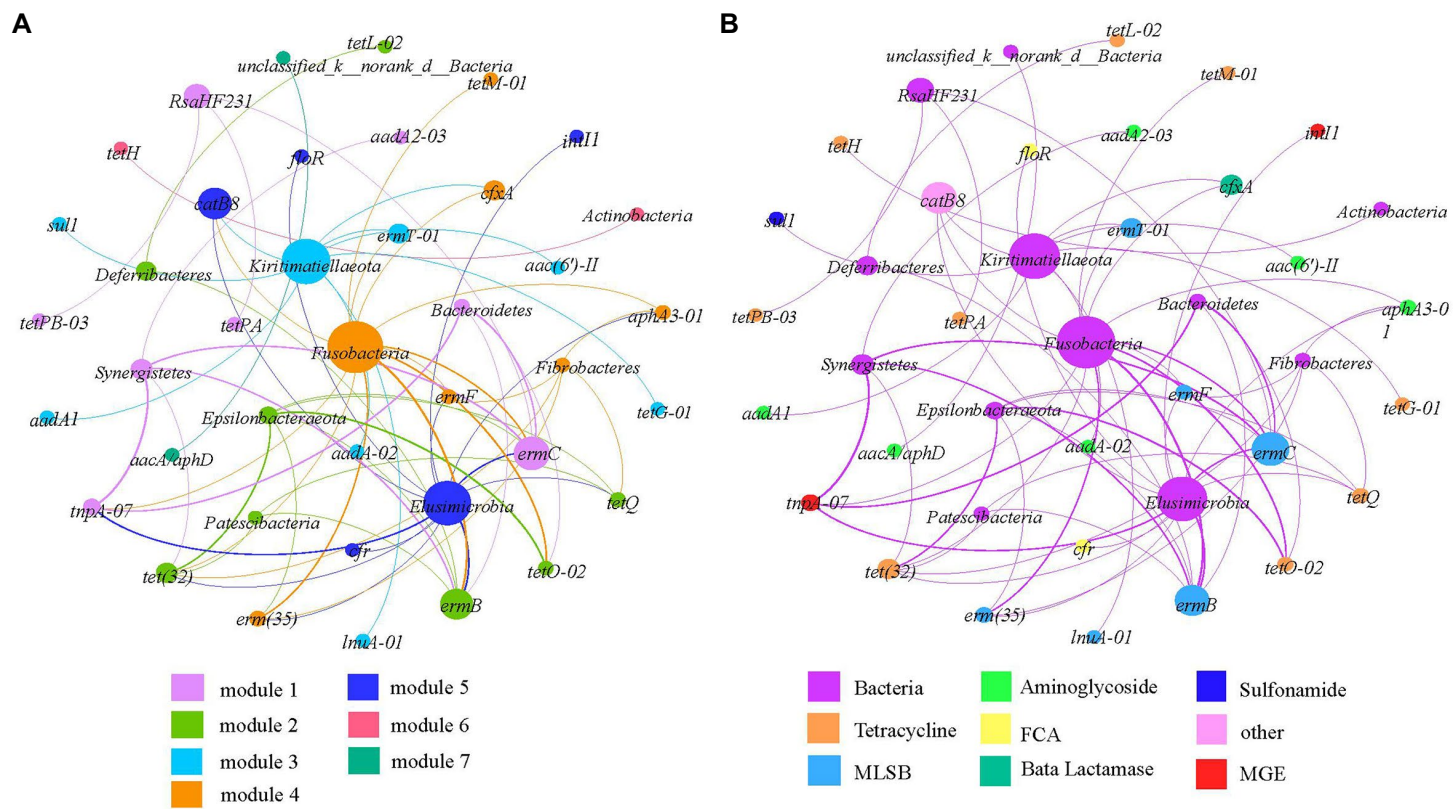
Network analysis revealing co-occurrence patterns among ARGs and bacterial taxa at the phylum level based on modularity taxa **(A)** and ARGs taxa **(B)**. The nodes coded with different colors represent different ARGs/MGEs and bacteria, and the edges correspond to strong and significant correlations between nodes. The size of each node is proportional to the number of significant correlations between nodes. The thickness of the edges is proportional to the correlation coefficient.

We conducted network analysis to explore the relationships among ARGs, MGEs, and the bacterial community and to identify the possible hosts of ARGs in complex environmental scenarios. ARGs/MGEs and the bacterial phylum/genera in the same module may co-occur under the same environmental pressure. We assumed that the non-random co-occurrence patterns between ARGs and microbial taxa could indicate the possible host information of ARGs if the ARGs and co-existing microbial phylum taxa possessed a strong and significant positive correlation (Spearman’s *ρ* > 0.6, *p* < 0.01). Results showed that the network consisted of 40 nodes and 63 edges and could be separated into seven modules (Figure 5A). The phylum Fusobacteria, Elusimicrobia, and Kiritimatiellaeota were hotspots in the ARG-bacteria network (Figure 5B). Fusobacteria might be co-occurring with the aminoglycosides resistance genes (*aph*A3-01), MLSB resistance genes [*erm*F, *erm*T-01, *erm*C, *erm*(35), and *erm*B], tetracycline resistance genes [*tet*(32), *tet*M-01, and *tet*O-02], β-Lactamase resistance genes (*cfx*A), and MGEs (*tnp*A-07); Elusimicrobia might be co-occurring with the aminoglycosides resistance genes (*aph*A3-01), MLSB resistance genes [*erm*F, *erm*(35), *erm*C, and *erm*B], tetracycline resistance genes [*tet*Q and *tet*(32)], other resistance genes (*cat*B8), and MGEs (*tnp*A-07 and *int*I1); Kiritimatiellaeota might be the potential host of aminoglycosides resistance genes [*aad*A1, *aac*(6′)-II and *aad*(A-02)], MLSB resistance genes (*erm*T-01 and *lun*A-01), tetracycline resistance genes (*tet*G-01), sulphonamides resistance genes (*sul*1), and β-Lactamase resistance genes (*cfx*A).

We further performed the correlation analysis between ARGs and bacterial taxa at the genus level to reveal the possible bacterial hosts. According to Figure 5A, 44 genus of bacteria showed significant correlation with multiple ARGs (Spearman’s *ρ* > 0.8, *p* < 0.01). The results are shown in Supplementary Figure S4A and reveal the network consisted of 88 nodes and 142 edges and could be separated into seven modules. The genus of *Mailhella*, *Flaviflexus*, *Chryseobacterium*, and *norank_f__Acidaminococcaceae* were prevalent in the co-occurrence network (Supplementary Figure S4B), and each contained ARGs conferring resistance to different classes of antibiotics. These bacterial genus were supposed to be potential hosts of aminoglycosides resistance genes (*aph*A3-01), β-lactamase resistance genes (*bla*TEM), tetracycline resistance genes [*tet*Q and *tet*(32)], MLSB resistance genes [*erm*F, *erm*C, *erm*B, and *erm*(35)], and MGEs (*int*I1, *tnp*A-07), which belonging to the phylum Proteobacteria, Actinobacteria, Bacteroidetes, and Firmicutes.

It was noteworthy that many pathogenic bacteria harbor the different ARGs. According to possible bacterial hosts, we only found that pathogenic bacteria *Helicobacter* may be potential hosts of *tet*Q (Spearman’s *ρ* > 0.8, *p* < 0.01). However, significant positive correlations were found between most of the ARGs and pathogenic bacterial *Bacteroides* (Supplementary Figure S5).

## Discussion

This work explored the ARG and MGE profile in free-range and intensive farms. Layers in the free-range farms were fed with grain with no antibiotics added. But the ARGs of manure samples covered almost all the major classes of antibiotics that are administered commonly to humans and animals (Selvam et al., 2012; Qian et al., 2016). According to a previous study, the prevalence of these ARGs in the layer manure was not likely driven by antibiotic selective pressure and is more likely a reflection of their baseline abundances in the environment (Liu et al., 2020). ARGs have always been present in the environment. ARGs that encode resistance for a large set of antibiotics have been found in 30,000-year-old Beringian permafrost and in bacteria isolated from prehistoric caves (D'Costa et al., 2011; Berglund, 2015). Tetracycline and sulphonamide genes have been reported as the most frequently detected ARGs in animal manures and livestock lagoons (Cheng et al., 2013; Guo et al., 2018). This is consistent with the result of the present study that *tnp*A-07, *sul*2, and *tet*M showed a high abundance. However, a study also found that the pollution of *sul*2, *tet*W, and *str*B were the worst in layer farms (Gu et al., 2020). The above differences may be due to the geographical environment or the type of antibiotics given. When comparing the relative abundance of all ARGs in the same antibiotic class, the total ARG abundance per class, as well as MGEs, was increasing with the increase of breeding scale, and the β-diversity differed significantly among layer manure samples from the five group farms. According to a previous study, the number of animals on farms explained differences in the ARG abundance in manure samples (Obeng et al., 2013). It is likely that the increase of breeding scale improved the infection of opportunistic pathogens among individuals. The use of antibiotics increased, and then the change of bacterial host caused the change of ARGs (Sun et al., 2016; Wang et al., 2021). In the present study, we have confirmed that ARGs and MGEs increased with the scale of breeding.

The tetracycline genes were the most frequently detected ARGs in the layer manure samples. This is consistent with the findings of studies quantifying ARGs in manure samples from poultry (Ji et al., 2012; Cheng et al., 2013), swine (Wang et al., 2017), and humans (Seville et al., 2009; Li et al., 2015). The MLSB resistance gene *erm*B was also found to be abundant in samples across intensive layer farms. Similar to the tetracycline genes, *erm*B was also detected in a wide range of bacterial species and associated with conjugative transposons (Roberts et al., 2012), which may explain the wide dissemination of the gene in the environment. The MLSB and tetracycline taxa of ARGs were significantly different among the manure samples across the five scales of breeding, indicating that the increase in breeding scale enriched ARGs to MLSB and tetracycline.

As for the MGEs, previous studies have indicated that the widespread nature of these genes is a proxy for the horizontal gene transfer potential of ARGs (Xiang et al., 2018). In previous studies, MGEs in animal manure were mainly *int*I1, with fewer reports on *tnp*A (Hsu et al., 2014; Ma et al., 2014); however, in the present study, *tnp*A-7 in layer manure was much more abundant than *int*I1, which may be related to the local environment and feeding mode. In the present study, the abundance of ARGs was highly correlated to the levels of MGEs detected in our layer samples based on linear regression analysis. The gene *sul*1 has a broad range of host bacteria for it is normally linked to integrins, and this study demonstrated a significant correlation between *sul*1 and *int*I1 (Skold, 2000; He et al., 2014). It has been suggested that antibiotic resistance can result from the acquiring of ARGs *via* horizontal gene transfer (Gaze et al., 2011; Zheng et al., 2017; Zhu et al., 2017a,b). Therefore, with the expansion of the breeding scale, the presence of potentially transferable ARGs in bacteria might play an important role in the dissemination of antibiotic resistance among populations (Martinez et al., 2015; Holmes et al., 2016).

According to the absolute abundance of 16S rRNA, there was no difference in the bacterial community abundance of layer manure samples from the five scale farms. However, the diversity in the bacterial community was significantly different among the manure samples in the five scale farms. The Shannon index of the bacteria community of the intensive layer manure samples was significantly higher than that of the free-range farms, and the Chao1 index of the layer manure samples of the layer farms with the scale of more than 10,000 was significantly higher than that of other layer farms.

Analysis of the microbial profile of the layer manure showed a dominance of phylum Firmicutes, Proteobacteria, Bacteroidetes, Actinobacteria, and Fusobacteria in the samples. At the genus level, *Lactobacillus*, *Ignatzschineria*, *Bacteriodes*, *Romboutsia*, and *Enterococcus* were the abundant taxa present in the layer gastrointestinal tract (Wei et al., 2013; Xiao et al., 2017), which is consistent with previous studies. However, the bacterial composition of the group N5000 was significantly different from that of other groups; the divergency might be explained by the differences between samples. In our study, manure material collected from the manure belt would have been exposed, and the manure microbiota could be affected by environmental factors, such as oxygen, temperature, and moisture (Wang et al., 2016). Some opportunistic pathogens were found in this study, and some dominant pathogens were significantly higher in the largest scale farms, indicating that the increase of the breeding scale led to the increase of the abundance of some pathogens.

Antibiotic resistance gene profiles have been reported to vary as a function of the taxonomic composition of bacterial communities, suggesting that the host range of ARGs is a major determinant of ARGs (Durso et al., 2012; Forsberg et al., 2014; Su et al., 2015). The present study and previous studies noticed that the development and dissemination of ARGs in feedlot environments could be influenced by production conditions, which can affect the evolution of the intestinal microbiome (He et al., 2014). The abundance and distribution of bacteria and antibiotic resistance may vary according to different rearing systems (Bojesen et al., 2003; El-Shibiny et al., 2005). The bacterial community composition plays a major role in the spread of ARGs, which involves bacterial host shifts (Su et al., 2015; Song et al., 2017; Liu et al., 2020). Network analysis at the phylum level found that fusobacteria, elusimicrobia, and kiritimatiellaeota were the main hosts of ARGs. The result was inconsistent with previous studies that bacterial phylum of Proteobacteria and Firmicutes had been recognized as the possible bacterial hosts (Yue et al., 2021). This may be due to the high abundance of Proteobacteria and Firmicutes (63.77–95.66%). Network analysis further elucidated the primary effects of the bacterial genus on ARGs by determining their potential host bacteria. The results showed that 30 genera were potential hosts of tetracycline resistance genes, with Proteobacteria and Firmicutes accounting for 72.9%; 20 genera were potential hosts of MLSB resistance genes, with Proteobacteria and Firmicutes accounting for 65.1%. The genes of *tet*(32), *erm*C, *tet*Q, *erm*B, and *tnp*A-07 showed high degree values in the co-occurrence network at the genus level, these tetracycline and MLSB genes were known to be present in a wide range of bacterial taxa (Aminov et al., 2001; Chopra and Roberts, 2001; Roberts et al., 2012; Song et al., 2017). This may explain why tetracycline and MLSB genes were significantly different among the layer manure samples of the five group farms. Besides, the high degree of *tnp*A-07 in layer manure samples was mainly due to horizontal gene transfer across bacterial taxa (Marathe et al., 2013; Na et al., 2021). These bacterial taxa have also been reported to be resistant to multiple antibiotics and able to capture new ARGs, which should receive more attention (Petrova et al., 2009; Yza et al., 2020). According to one study, a significant positive correlation was found between most of the ARGs and between most of the bacterial genera, suggesting the co-abundance relationship between the ARGs and between the bacterial genera (Xie et al., 2019). In this study, significant positive correlations were found between most of the ARGs and pathogenic bacterial *Bacteroides*. This showed that *Bacteroides* may play an important role in the spread of ARGs and that they are dangerous to the breeding industry. However, the correlation between pathogenic bacteria and most ARGs significantly indicated that the pathogenic bacteria in this study may not be potential hosts of ARGs.

One of the major limitations of this study is that there is fewer sample numbers and samples from only layer farms. Further, another limitation is that environmental factors were not measured and therefore the impact of environmental factors on ARGs cannot be quantified. Additional studies are needed to investigate various types of farms and environmental conditions. Future studies, and existing knowledge likely to help in improving management of farms to reduce the pollution of ARGs.

## Conclusion

By investigating the layer manure of one free-range and four intensive farms with different breeding scales, we found that the pollution of ARGs in intensive layer farms was significantly higher than that in the free-range layer farms. With the increase of breeding scale, the abundance of the bacteria community did not show a significant difference, but the pollution of ARGs and some pathogen genera gradually increased. Moreover, increasing layer breeding scale led to greater diversity in the bacterial community. We hypothesized that increased bacterial diversity might have contributed to the spread of ARGs. Therefore, considering the potential impact of ARG pollution on human health, it is recommended to cultivate about 10,000 layers in single-shed of a farm to control the spread of ARGs in the environment.

## Data Availability Statement

The datasets presented in this study can be found in online repositories. The names of the repository/repositories and accession number(s) can be found at: https://www.ncbi.nlm.nih.gov/, SRR16607894.

## Author Contributions

LW: methodology, data curation, visualization, investigation, validation, and writing—review and editing. BC: supervision, writing—review and editing, and validation. All authors contributed to the article and approved the submitted version.

## Funding

This research was funded by National Natural Science Foundation of China, grant number 31772450.

## Conflict of Interest

The authors declare that the research was conducted in the absence of any commercial or financial relationships that could be construed as a potential conflict of interest.

## Publisher’s Note

All claims expressed in this article are solely those of the authors and do not necessarily represent those of their affiliated organizations, or those of the publisher, the editors and the reviewers. Any product that may be evaluated in this article, or claim that may be made by its manufacturer, is not guaranteed or endorsed by the publisher.

## Abbreviations

ARGs: Antibiotic resistance genes
MLSB: Macrolide-lincosamide-streptogramin B
MGEs: Mobile genetic elements
FCAs: Fluoroquinolones, quinolones, florfenicols, chloramphenicols, and amphenicols
NMDS: Non-metric multidimensional scaling
ANOSIM: Analysis of similarities.
