# Supplementary material for: Fate of Antibiotic Resistance Genes and Changes in Bacterial Community With Increasing Breeding Scale of Layer Manure
Source: Front Microbiol. 2022 Mar 9;13:857046. doi: 10.3389/fmicb.2022.857046 (PMC8959713; doi:10.3389/fmicb.2022.857046)
Supplement: Supplementary file 1 [file Table_1.docx]

Table S1 Sequence of primers of ARGs and MGEs

| **Genes** | **Forward Primer** | **Reverse Primer** |
| --- | --- | --- |
| *aac*(6')-IB-1 | GTTTGAGAGGCAAGGTACCGTAA | GAATGCCTGGCGTGTTTGA |
| *aac*(6')-II | CGACCCGACTCCGAACAA | GCACGAATCCTGCCTTCTCA |
| *aac*A/*aph*D | AGAGCCTTGGGAAGATGAAGTTT | TTGATCCATACCATAGACTATCTCATCA |
| *aad*A-02 | CGAGATTCTCCGCGCTGTA | GCTGCCATTCTCCAAATTGC |
| *aad*A1 | AGCTAAGCGCGAACTGCAAT | TGGCTCGAAGATACCTGCAA |
| *aad*A2-03 | CAATGACATTCTTGCGGGTATC | GACCTACCAAGGCAACGCTATG |
| *aad*D | CCGACAACATTTCTACCATCCTT | ACCGAAGCGCTCGTCGTATA |
| *aad*E | TACCTTATTGCCCTTGGAAGAGTTA | GGAACTATGTCCCTTTTAATTCTACAATCT |
| *aph*A1 | TGAACAAGTCTGGAAAGAAATGCA | CCTATTAATTTCCCCTCGTCAAAAA |
| *aph*A3-01 | CGGAATTGAAAAAACTGATCGAA | ATACCGGCTGTCCGTCATTT |
| *bla*CTX-M-02 | GGAGGCGTGACGGCTTTT | TTCAGTGCGATCCAGACGAA |
| *bla*OXA1/*bla*OXA30 | CGGATGGTTTGAAGGGTTTATTAT | TCTTGGCTTTTATGCTTGATGTTAA |
| *bla*OXA10-01 | CGCAATTATCGGCCTAGAAACT | TTGGCTTTCCGTCCCATTT |
| *bla*PSE | TTGTGACCTATTCCCCTGTAATAGAA | TGCGAAGCACGCATCATC |
| *bla*TEM | AGCATCTTACGGATGGCATGA | TCCTCCGATCGTTGTCAGAAGT |
| *cat*B3 | GGTCATGCCCGGAATCAAGA | CGATAGCGTAAGGCTCCACA |
| *cat*B8 | CACTCGACGCCTTCCAAAG | CCGAGCCTATCCAGACATCATT |
| *cfr* | GCAGGTTGGGAGTCATTTTG | ACGGTTGGCTAGAGCTTCAC |
| *cfx*A | TCATTCCTCGTTCAAGTTTTCAGA | TGCAGCACCAAGAGGAGATGT |
| *dfr*A1 | GGAATGGCCCTGATATTCCA | AGTCTTGCGTCCAACCAACAG |
| *erm*(35) | TTGAAAACGATGTTGCATTAAGTCA | TCTATAATCACAACTAACCACTTGAACGT |
| *erm*B | TAAAGGGCATTTAACGACGAAACT | TTTATACCTCTGTTTGTTAGGGAATTGAA |
| *erm*C | TTTGAAATCGGCTCAGGAAAA | ATGGTCTATTTCAATGGCAGTTACG |
| *erm*F | CAGCTTTGGTTGAACATTTACGAA | AAATTCCTAAAATCACAACCGACAA |
| *erm*T-01 | CATATAAATGAAATTTTGAG | ACGATTTGTATTTAGCAACC |
| *flo*R | ATTGTCTTCACGGTGTCCGTTA | CCGCGATGTCGTCGAACT |
| *lnu*A-01 | TGACGCTCAACACACTCAAAAA | TTCATGCTTAAGTTCCATACGTGAA |
| *lnu*B-01 | TGAACATAATCCCCTCGTTTAAAGAT | TAATTGCCCTGTTTCATCGTAAATAA |
| *mat*A/*mel* | TAGTAGGCAAGCTCGGTGTTGA | CCTGTGCTATTTTAAGCCTTGTTTCT |
| *mef*A | CCGTAGCATTGGAACAGCTTTT | AAACGGAGTATAAGAGTGCTGCAA |
| *str*B | GCTCGGTCGTGAGAACAATCT | CAATTTCGGTCGCCTGGTAGT |
| *sul*1 | CACCGGAAACATCGCTGCA | AAGTTCCGCCGCAAGGCT |
| *sul*2 | CTCCGATGGAGGCCGGTAT | GGGAATGCCATCTGCCTTGA |
| *tet*(32) | CCATTACTTCGGACAACGGTAGA | CAATCTCTGTGAGGGCATTTAACA |
| *tet*(36)-01 | AGAATACTCAGCAGAGGTCAGTTCCT | TGGTAGGTCGATAACCCGAAAAT |
| *tet*B-01 | GCCCAGTGCTGTTGTTGTCAT | TGAAAGCAAACGGCCTAAATACA |
| *tet*G-01 | TCAACCATTGCCGATTCGA | TGGCCCGGCAATCATG |
| *tet*H | TTTGGGTCATCTTACCAGCATTAA | TTGCGCATTATCATCGACAGA |
| *tet*L-02 | ATGGTTGTAGTTGCGCGCTATAT | ATCGCTGGACCGACTCCTT |
| *tet*M-01 | CATCATAGACACGCCAGGACATAT | CGCCATCTTTTGCAGAAATCA |
| *tet*O-02 | CAACATTAACGGAAAGTTTATTGTATACCA | TTGACGCTCCAAATTCATTGTATC |
| *tet*PA | AGTTGCAGATGTGTATAGTCGTAAACTATCTATT | TGCTACAAGTACGAAAACAAAACTAGAA |
| *tet*PB-03 | TGGGCGACAGTAGGCTTAGAA | TGACCCTACTGAAACATTAGAAATATACCT |
| *tet*Q | CGCCTCAGAAGTAAGTTCATACACTAAG | TCGTTCATGCGGATATTATCAGAAT |
| *tet*R-03 | CGCGATGGAGCAAAAGTACAT | AGTGAAAAACCTTGTTGGCATAAAA |
| *tet*T | CCATATAGAGGTTCCACCAAATCC | TGACCCTATTGGTAGTGGTTCTATTG |
| *tet*W-01 | ATGAACATTCCCACCGTTATCTTT | ATATCGGCGGAGAGCTTATCC |
| *tet*X | AAATTTGTTACCGACACGGAAGTT | CATAGCTGAAAAAATCCAGGACAGTT |
| *tnp*A-07 | TGCAGATGGTTTAACCTTGGATATTT | TCGGTTCATCAAACTGCTTCAC |
| *int*I1 | CGAACGAGTGGCGGAGGGTG | TACCCGAGAGCTTGGCACCCA |

Table S2 List of classifications of ARGs and MGEs genes

| Taxa | Gene | Taxa | Gene |
| --- | --- | --- | --- |
| Aminoglycoside | *aac*(6')-IB-1 | Tetracycline | *tet*(32) |
|  | *aac*(6')-II |  | *tet*(36)-01 |
|  | *aac*A/aphD |  | *tet*B-01 |
|  | *aad*A-02 |  | *tet*G-01 |
|  | *aad*A1 |  | *tet*H |
|  | *aad*A2-03 |  | *tet*L-02 |
|  | *aad*D |  | *tet*M-01 |
|  | *aad*E |  | *tet*O-02 |
|  | *aph*A1 |  | *tet*PA |
|  | *aph*A3-01 |  | *tet*PB-03 |
|  | *str*B |  | *tet*Q |
| MLSB | *erm*(35) |  | *tet*R-03 |
|  | *erm*B |  | *tet*T |
|  | *erm*C |  | *tet*W-01 |
|  | *erm*F |  | *tet*X |
|  | *erm*T-01 | Bata Lactamase | *bla*CTX-M-02 |
|  | *lnu*A-01 |  | *bla*OXA1/*bla*OXA30 |
|  | *lnu*B-01 |  | *bla*OXA10-01 |
|  | *mat*A/*mel* |  | *bla*PSE |
|  | *mef*A |  | *bla*TEM |
| FCA | *cat*B3 |  | *cfx*A |
|  | *cfr* | other | *cat*B8 |
|  | *flo*R |  | *dfr*A1 |
| Sulfonamide | *sul*1 | MGE | *tnp*A-07 |
|  | *sul*2 |  | *int*I1 |

Table S3 Differences of ARGs/MGEs abundance between layer manure samples

|  | CK | N500 | N5000 | N10000 | N20000 |
| --- | --- | --- | --- | --- | --- |
| Aminoglycoside | 0.0551±0.0323 b | 0.1271±0.0518 b | 0.4477±0.0845 ab | 0.5902±0.2055 ab | 0.7171±0.2693 a |
| Bata Lactamase | 0.0106±0.0068 a | 0.0125±0.0064 a | 0.0189±0.0047 a | 0.0335±0.0111 a | 0.0303±0.0106 a |
| FCA | 0.0656±0.0334 b | 0.1395±0.0528 b | 0.4666±0.0891 ab | 0.6238±0.2164 ab | 0.7474±0.2799 a |
| MLSB | 0.0381±0.0022 c | 0.167±0.0361 bc | 0.1549±0.0033 bc | 0.381±0.1136 ab | 0.5268±0.1484 a |
| Sulfonamide | 0.0157±0.0099 b | 0.0193±0.0103 b | 0.1279±0.0351 ab | 0.113±0.0452 ab | 0.1469±0.0582 a |
| Tetracycline | 0.3942±0.043 bc | 0.3801±0.0246 bc | 0.3036±0.0265 c | 0.5507±0.1301 b | 0.9043±0.0774 a |
| Other/efflux | 0.0003±0.0003 a | 0.0058±0.0041 a | 0.0379±0.0139 a | 0.0253±0.013 a | 0.0407±0.0187 a |
| total ARGs | 0.5796±0.0899 c | 0.8512±0.1312 c | 1.5574±0.2515 bc | 2.3176±0.6817 ab | 3.1134±0.6472 a |
| *int*I1 | 0.0002±0.0001 a | 0.0000±0.0000 a | 0.0000±0.0000 a | 0.0015±0.0006 a | 0.0017±0.0011 a |
| *tnp*A-07 | 0.0113±0.0057 b | 0.0225±0.0106 b | 0.0434±0.0031 b | 0.0803±0.0174 b | 0.3119±0.0812 a |
| total MGEs | 0.0114±0.0055 b | 0.0225±0.0106 b | 0.0434±0.0031 b | 0.0818±0.018 b | 0.3136±0.0331 a |

Table S4 Pearson correlations analysis between ARGs and MGEs

|  | tnpA07 | | intI1 | |  | tnpA07 | | intI1 | |
| --- | --- | --- | --- | --- | --- | --- | --- | --- | --- |
|  | coefficient | *P* | coefficient | *P* |  | coefficient | *P* | coefficient | *P* |
| *aac*(6')-IB-1 | **0.593** | **0.020** | 0.367 | 0.178 | *erm*T-01 | **0.712** | **0.003** | 0.131 | 0.642 |
| *aac*(6')-II | **0.604** | **0.017** | 0.368 | 0.177 | *flo*R | 0.509 | 0.053 | 0.397 | 0.142 |
| *aac*A/*aph*D | 0.346 | 0.207 | 0.427 | 0.112 | *lnu*A-01 | **0.882** | **0.000** | -0.027 | 0.924 |
| *aad*A-02 | **0.615** | **0.015** | 0.351 | 0.199 | *lnu*B-01 | 0.12 | 0.670 | 0.21 | 0.452 |
| *aad*A1 | **0.6** | **0.018** | 0.274 | 0.323 | *mat*A/*mel* | 0.187 | 0.505 | 0.206 | 0.461 |
| *aad*A2-03 | **0.606** | **0.017** | 0.348 | 0.204 | *mef*A | 0.304 | 0.270 | 0.311 | 0.260 |
| *aad*D | -0.002 | 0.993 | 0.111 | 0.694 | *str*B | 0.513 | 0.051 | 0.364 | 0.182 |
| *aad*E | 0.213 | 0.446 | 0.322 | 0.241 | *sul*1 | 0.49 | 0.063 | **0.87** | **0.000** |
| *aph*A1 | -0.016 | 0.955 | -0.055 | 0.845 | *sul*2 | 0.377 | 0.166 | 0.301 | 0.276 |
| *aph*A3-01 | 0.174 | 0.535 | 0.499 | 0.059 | *tet*(32) | 0.378 | 0.165 | **0.834** | **0.000** |
| *bla*CTX-M-02 | -0.198 | 0.479 | 0.042 | 0.882 | *tet*(36)-01 | 0.270 | 0.330 | 0.327 | 0.234 |
| *bla*OXA1/*bla*OXA30 | 0.384 | 0.157 | 0.133 | 0.637 | *tet*B-01 | -0.030 | 0.917 | 0.157 | 0.576 |
| *bla*OXA10-01 | 0.347 | 0.205 | 0.25 | 0.369 | *tet*G-01 | 0.473 | 0.075 | 0.355 | 0.194 |
| *bla*PSE | 0.412 | 0.127 | 0.346 | 0.207 | *tet*H | 0.101 | 0.72 | -0.156 | 0.579 |
| *bla*TEM | 0.099 | 0.726 | 0.488 | 0.065 | *tet*L-02 | **0.891** | **0.000** | 0.089 | 0.753 |
| *cat*B3 | 0.516 | 0.049 | 0.374 | 0.169 | *tet*M-01 | **0.825** | **0.000** | 0.08 | 0.777 |
| *cat*B8 | 0.472 | 0.076 | 0.386 | 0.155 | *tet*O-02 | 0.367 | 0.178 | **0.782** | **0.001** |
| *cfr* | 0.169 | 0.548 | **0.536** | **0.040** | *tet*PA | 0.169 | 0.548 | **0.717** | **0.003** |
| *cfx*A | 0.364 | 0.182 | 0.318 | 0.247 | *tet*PB-03 | 0.054 | 0.847 | **0.583** | **0.022** |
| *dfr*A1 | 0.376 | 0.168 | 0.104 | 0.711 | *tet*Q | 0.029 | 0.920 | 0.358 | 0.190 |
| *erm*(35) | 0.186 | 0.508 | 0.351 | 0.200 | *tet*R-03 | -0.069 | 0.807 | 0.141 | 0.616 |
| *erm*B | **0.877** | **0.000** | 0.314 | 0.255 | *tet*T | 0.091 | 0.747 | 0.095 | 0.736 |
| *erm*C | **0.538** | **0.038** | **0.632** | **0.011** | *tet*W-01 | -0.229 | 0.411 | -0.073 | 0.795 |
| *erm*F | 0.228 | 0.414 | 0.509 | 0.053 | *tet*X | 0.209 | 0.454 | 0.412 | 0.127 |


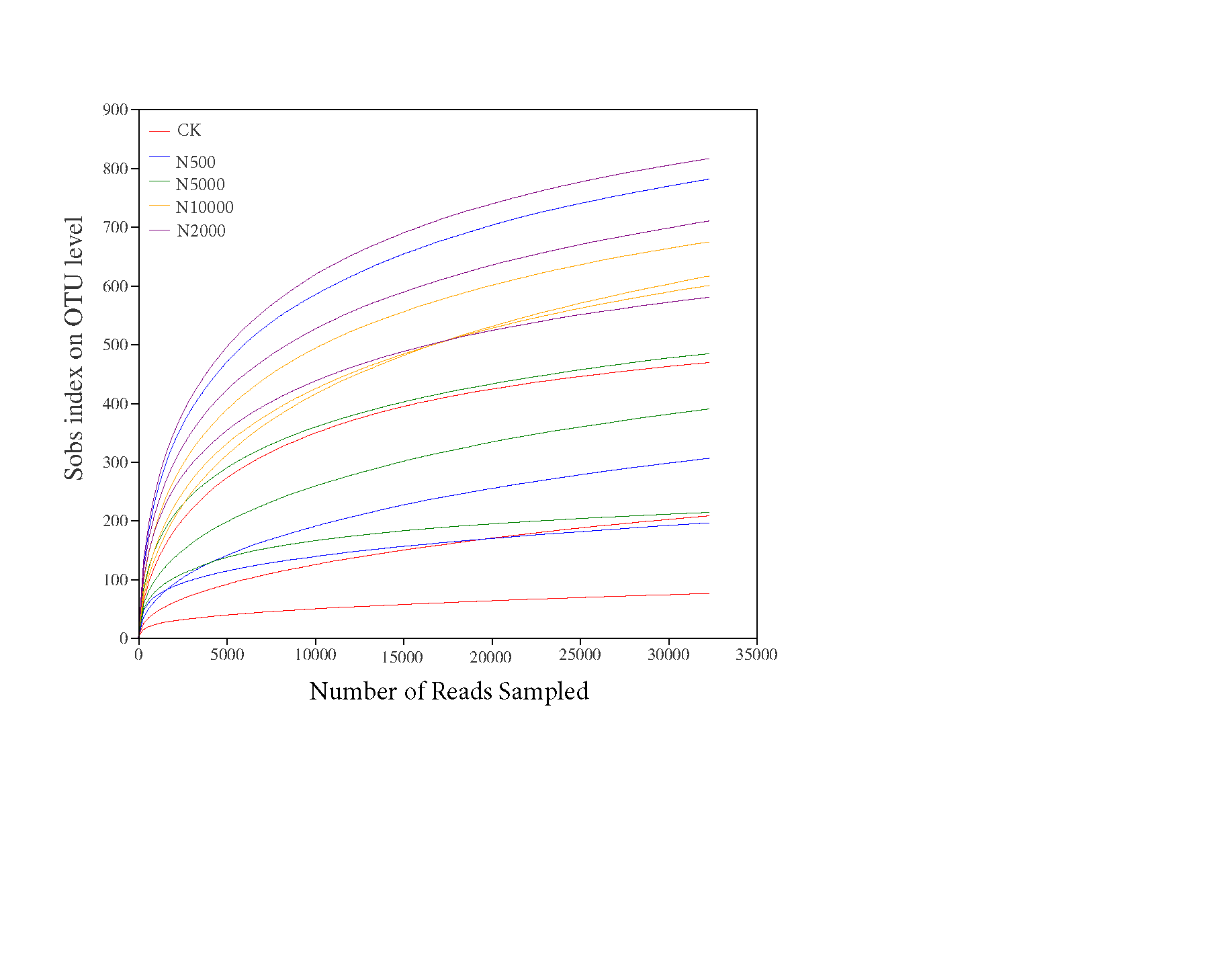


Figure S1. The rarefaction curve of bacterial communities in layer manure samples.

Figure S2. The absolute abundance of 16SrRNA genes at different layer manure samples. Different lowercase letters indicated significant differences (ANOVA,*P* < 0.05) between manure samples.


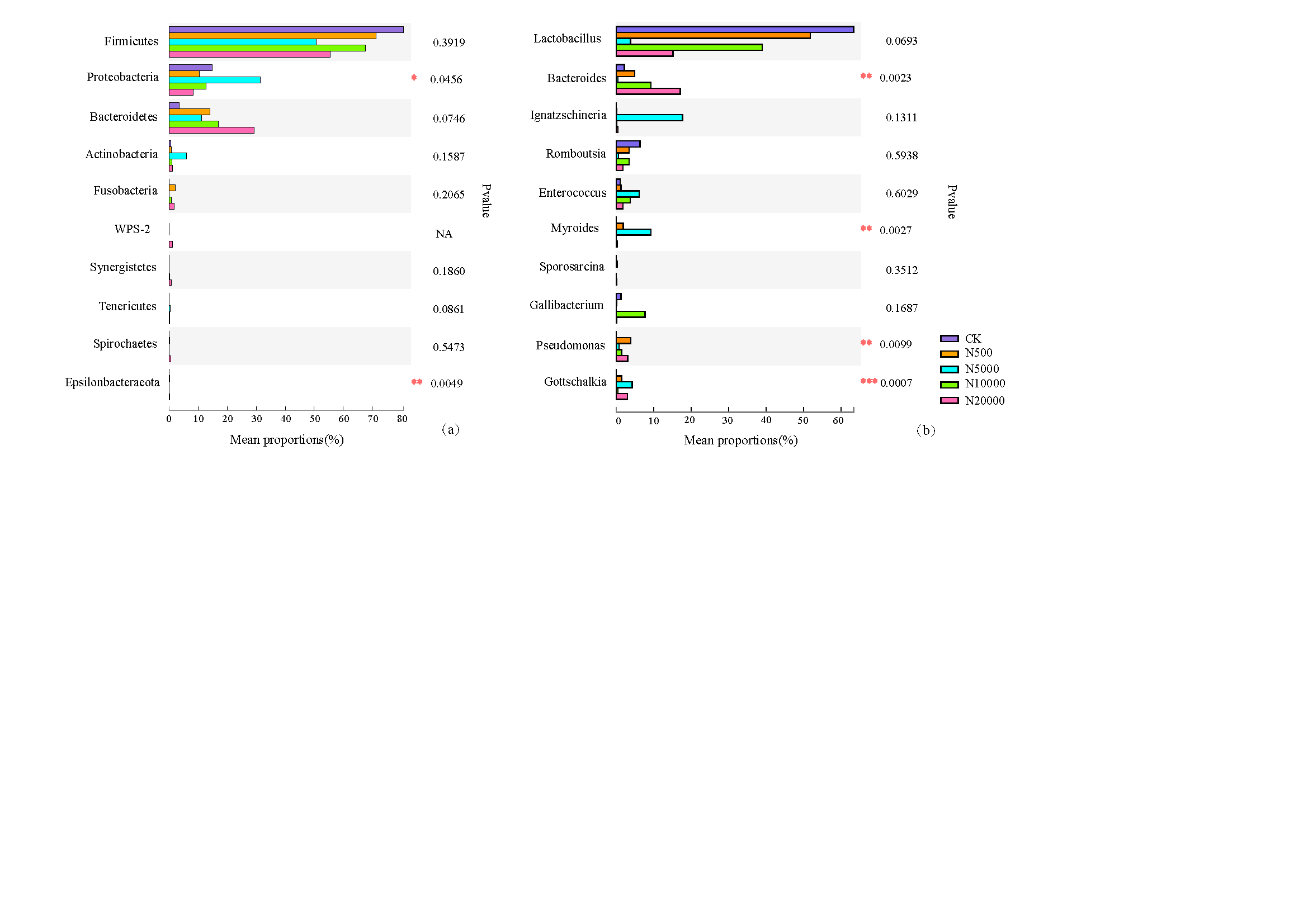


Figure S3. Differences in the relative abundance of the dominant bacterial phylum(a) and genus level between layer manure samples.

*** indicate P < 0.001; ** indicate P <0 .01; * indicate P <0 .05.


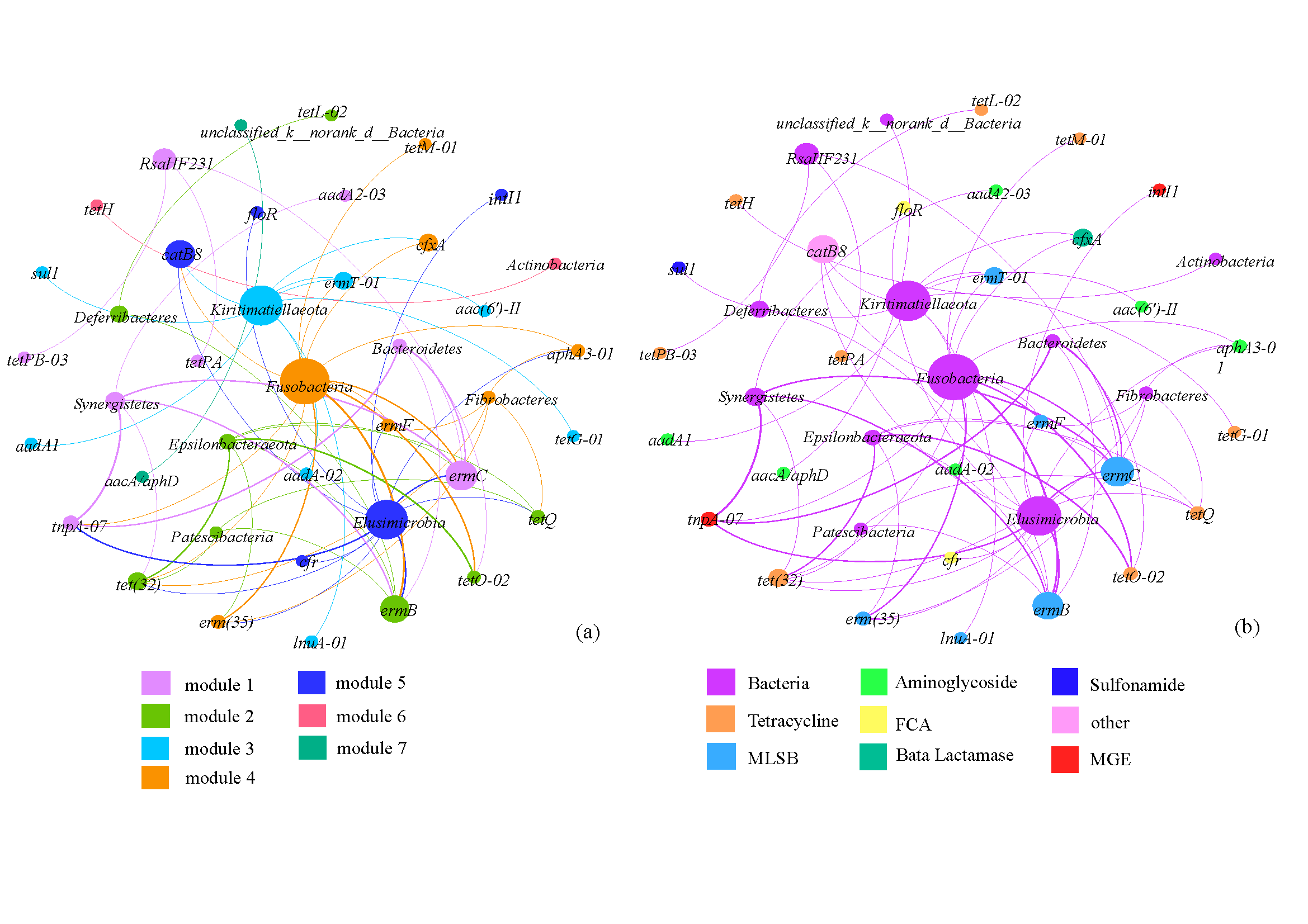


Figure S4. Network analysis revealing co-occurrence patterns among antibiotic resistance genes(ARGs) and bacterial taxa at the genus level based on modularity taxa(a) and ARGs taxa. The nodes coded with different colors represent different ARGs/MGEs and bacteria, and the edges correspond to strong and significant correlations between nodes. The size of each node is proportional to the number of significant correlations between nodes. The thickness of the edges is proportional to the correlation coefficient.


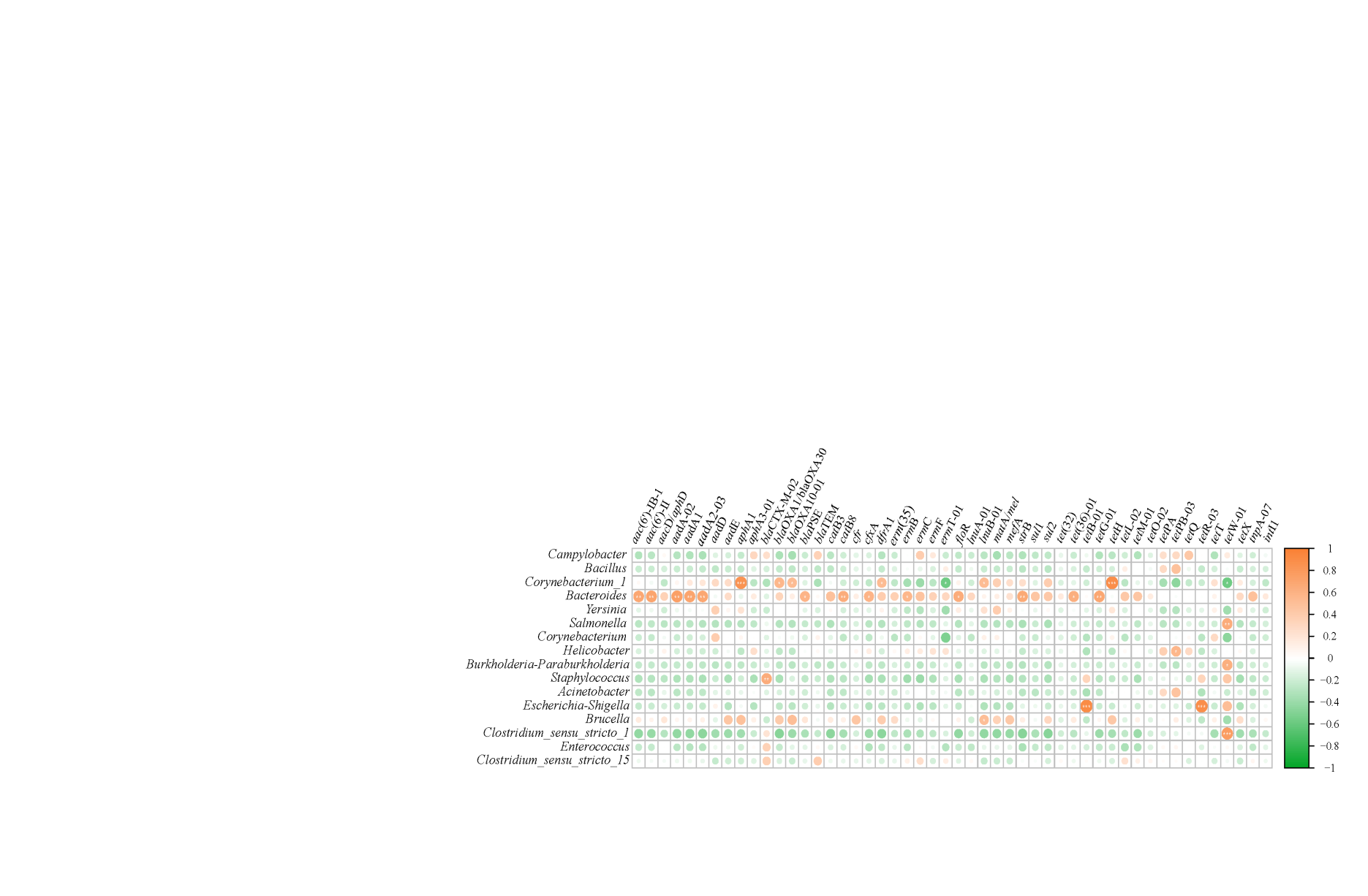


Figure S5. The pearson correlation between pathogenic bacterial genera and ARGs.
